# Oxalate Contents of Raw, Boiled, Wok-Fried and Pesto and Juice Made from Fat Hen (*Chenopodium album*) Leaves

**DOI:** 10.3390/foods8010002

**Published:** 2018-12-21

**Authors:** Geoffrey Savage, Leo Vanhanen

**Affiliations:** Department of Food, Wine and Molecular Biosciences, Faculty of Agriculture and Life Sciences, Lincoln University, Lincoln 7647, Canterbury, New Zealand; leo.vanhanen@lincoln.ac.nz

**Keywords:** total, soluble and insoluble oxalates, calcium, boiling, wok frying, pesto, juicing

## Abstract

The total, soluble, and insoluble oxalate contents of fresh and wok-fried fat hen (*Chenopodium album*) leaves were extracted and measured using High pressure liquid chromatography. The total oxalate content of the raw leaves was 1112.4 mg/100 g dry matter (DM), and the levels were significantly reduced by boiling (682.8 mg/100 g DM) or cooking the leaves in a wok (883.6 mg/100 g DM). The percentages of soluble oxalate contents in the total oxalates of the raw and boiled leaves were similar (mean 75%), while the proportion of soluble oxalate content in the wok-fried leaves was reduced to 53.4% of the total, giving a significant increase in the insoluble oxalate content of the wok-fried leaves. The percentage of insoluble calcium in the total calcium was significantly reduced (*p* < 0.05) when the leaves were boiled, but the insoluble oxalate content significantly increased (67.2%) in the wok-fried leaves when compared to the content of the original raw leaves. Processing the cooked leaves into pesto or extracting the juice gave final products that contained significantly reduced total and soluble oxalate contents. The addition of calcium chloride to the juice caused a very small reduction in the soluble oxalate content in the juice.

## 1. Introduction

Fat hen (*Chenopodium album* L.) is a fast-growing, weedy annual plant found in many parts of the world. Its common names include goosefoot and lamb’s quarters. It is extensively cultivated and consumed in Northern India as a food crop where its Hindi name is bathua. The leaves and young shoots of this plant are used in dishes—such as soups, curries, and paratha-stuffed breads—and are especially popular in the Punjab. Elsewhere in the world, the plant is considered to be a weed and is rarely eaten. However, the leaves and young shoots may be eaten as a vegetable, either steamed or cooked like spinach. Evidence that the plant has been used as a food plant for centuries was confirmed by the occurrence of carbonized plant remains in storage pits and ovens at Iron Age, Viking Age, and Roman sites in Europe, where seeds have been found mixed with conventional grains and even inside the stomachs of Danish bog bodies [1].

In many developing countries, vegetables are a reliable and rich source of minerals and nutrients, especially for people who adopt a vegetarian lifestyle. It is also important to note that in developed countries a plant or herb can be rediscovered and promoted as a new ‘super food’. In this case, it is important to have a record of its nutrient contents and any possible antinutrients that may cause adverse metabolic effects when eaten or may interfere with the absorption of other important nutrients from the leaves. Fat hen is classified as a Chenopodiaceae, and plants in this family are well known to contain high levels of oxalates.

Oxalate is not an essential nutrient for humans and, if possible, it should not be consumed in large amounts. However, oxalates are found in many kinds of edible plants in varying concentrations [2,3] and, if consumed in large amounts, they may be harmful to human health [2]. Oxalates occur in two forms in plants: water soluble oxalate (bound to Na^+^ or K^+^) or water insoluble oxalate (bound to divalent ions, such as Ca^2+^ and Mg^2+^). An intake of large amounts of soluble oxalate can increase the risk of kidney stone development in susceptible people because of the increased concentration of oxalate in the urine. A large increase in the consumption of oxalates in the diet will increase the risk of kidney stone development, so it is important to identify high oxalate-containing foods and, if possible, reduce these levels by processing [3]. In addition, the binding of soluble oxalate to calcium in the intestine may reduce calcium intakes to lower than recommended levels if the diet does not include adequate calcium sources. 

The first report of the total oxalate content of fat hen leaves ranged from 361–2027 mg/100 g dry matter (DM) (mean 1100 mg/100 g DM) for plants grown in Spain [4]. This was followed by reported values of 1099 mg total oxalate/100 g dry leaves found growing in Almeria, Southeast Spain [5]. Raghuvanshi et al. [6] reported a value of 883.1 mg total oxalate/100 g DM for fat hen found growing as a weed in wheat fields in Pantnagar, India. Fresh fat hen leaves grown in Tamil Nadu, India, contained 539.9 and 375.8 mg/100 g DM for total and soluble oxalate, while cooked leaves contained 545.7 and 377.5 mg/100 g DM for the same [6]. 

In developing countries, green leafy vegetables such as fat hen are reliable and important sources of minerals, particularly calcium, and the total calcium in raw and cooked fat hen leaves was 539.9 and 545.7 mg/100 g DM, respectively [7]. Amarial and Pius [7] went on to measure the bioavailability of calcium using a simulated gastric equilibrium dialysis analysis after a simulated in vitro gastric digestion. The mean bioavailability of calcium of the raw and cooked leaves was 32.9%. This was a relatively low value compared with the other green leafy vegetables measured in this study. The low bioavailability observed in this study could be explained by the high content of insoluble oxalate in the leaves. It has been observed that insoluble oxalate is largely made up of calcium oxalate [8], and that the calcium oxalate molecule is 31.3% calcium. Therefore, a large amount of the total calcium content in the leaves of fat hen could be unavailable because of being bound to the insoluble oxalate in leaf tissues.

The addition of food ingredients containing a high calcium content to foods containing high soluble oxalate contents has been shown to significantly reduce the soluble oxalate content of the final product [9]. Baking taro leaves with milk [10,11] and with spinach leaves [12] has also been shown to be effective. The addition of four different calcium salts during the preparation of a green juice made from spinach leaves was also very effective at reducing the soluble oxalate content of the final juice [12]. The preparation of pesto and a juice from fat hen also offers the possibility of reducing the soluble oxalate of the final product by the addition of calcium-containing ingredients. 

The objective of this study was to investigate the oxalate compositions of raw and cooked leaves of fat hen harvested in New Zealand. In addition, the study investigated the oxalate content of a pesto made from processing the leaves at room temperature and the preparation of a mixed juice using the raw leaves.

## 2. Materials and Methods

### 2.1. Source of Materials and Preparation

Fat hen (*Chenopodium album* L.) plants were grown in a Wakanui silt loam soil at the Horticulture Research Area, Lincoln University, Canterbury, New Zealand (43°38′52″ S, 172°28′0.5″ E) at 19 m above sea level. The Wakanui silt loam had a good base fertility and no manure or chemical fertilizers were used. The plants were irrigated as required. Approximately 1.5 kg of leaves were harvested on 15 February 2018 when the plants had reached a height of 150 mm. The leaves were cut into 1–2 mm pieces with a stainless-steel knife prior to sampling.

### 2.2. Boiling

An approximately 200-g sample of fresh leaves was added to 400 mL of boiling tap water in a saucepan. The mixture was stirred and allowed to boil for two min. The cooked leaves were then poured into a colander and allowed to drain for two min to remove the excess moisture.

### 2.3. Wok-Frying

Approximately 200 g fresh leaves were added to 200 mL of heated (170 °C) canola oil (Pams salad and cooking oil, Pams Products Ltd., Roskill, Auckland, New Zealand) and gently stirred while cooking for 2 min. The mixture was then removed from the steel wok using a slotted spoon and placed on absorbent kitchen paper to cool for 2 min to allow the excess oil to be removed from the leaves.

### 2.4. Pesto

The pesto was made from freshly harvested leaves. The vegetables and fruit, purchased from the New World Supermarket, Lincoln, Canterbury, New Zealand (Table 1), were chopped with a stainless-steel knife and processed in a Breville bottom-drive food processor (Briscoes Homeware Ltd., Auckland, New Zealand). The mixture was processed at full speed until it formed a fine paste that was then stored at −20 °C until analysis could take place.

### 2.5. Juice

All vegetables and fruits, except the fat hen leaves, were purchased fresh from the New World Supermarket, Lincoln, Canterbury, NZ in April 2016. The fruits and vegetables were trimmed of non-edible parts using a stainless-steel knife and the remaining edible portions were chopped, weighed, and processed with the fresh fat hen leaves using a masticating juicer (Oscar 9000, Dongah Industrial Co., Ltd, Gyeongsangnam Do, South Korea) following the recipe shown in Table 2. A calcium source—200 mg calcium chloride (96% CaCl_2_, Kirsch Phama GmbH., Salzgitter, Germany)—was added to 100 mL of the juice, and the juice was homogenized using a blender (NutriBullet, NBR-1207M, 600 W, Homeland Housewares LLC, Los Angeles, CA, USA).

### 2.6. Dry Matter

The dry matter contents of each sample of the fresh, boiled, and wok-fried leaves were determined by drying in an oven (Watvic, Watson Victor Ltd., Auckland, NZ) to a constant weight at 105 °C [13].

### 2.7. Extraction of Total and Soluble Oxalic Acids

The measurement of total and soluble oxalates followed the method outlined by Savage et al. [3]. Four replicates of 5 g from each sample of raw or cooked fat hen leaves and the extracted juice and pesto were used to measure the total oxalate contents, and four 5 g replicates were extracted to measure the soluble oxalate contents. Forty mL of 0.2 M HCl (Aristar, BDH Chemicals, Ltd., Poole, Dorset, UK) were added to conical flasks for the total oxalate extraction, and 40 mL of high purity water (18.2 MΩ·cm, Barnstead International, Dubuque, IA, USA,) were added for the extraction of soluble oxalates. All flasks were placed in an 80 °C shaking water bath for 20 min. The solutions were then quantitatively transferred into volumetric flasks, allowed to cool, and then made up to 100 mL with 0.2 M HCl and high purity water, respectively.

The extracts in the volumetric flasks were filtered through a cellulose acetate syringe filter with a pore size of 0.45 µm (dismic-25cs, Advantec, Milpitas, CA, USA) into 1 mL glass vials. The samples were analyzed with a high performance liquid chromatography (HPLC) system using a 300 mm × 7.8 mm Rezex ion exclusion column (Phenomenex Inc., Torrance, CA, USA) attached to a cation-H guard column (Bio-Rad, Richmond, CA, USA) held at 25 °C. Analysis was performed by injecting 20 µL of sample or standard onto the column using an aqueous solution of 25 mM sulfuric acid (HPLC grade, Baker Chemicals, Phillipsburg, NJ, USA) as the mobile phase, then pumped isocratically at 0.6 mL/min, with the peaks detected at 210 nm. The HPLC equipment consisted of a Shimadzu LC-10AD pump, CTO-10A column oven, SPD-10Avp UV-Vis detector (Shimadzu, Kyoto, Japan), and a Waters 717 plus auto-sampler (Waters, Milford, MA, USA). Data acquisition and processing were undertaken using a PeakSimple Chromatography Data System (model 203, SRI Instruments, Torrance, CA, USA) and PeakSimple software version 4.37 (SRI Instruments, Torrance, CA, USA). The oxalic acid peak was identified by comparing the retention time to a standard solution and by spiking an already-filtered sample containing a known amount of oxalic acid standard. The insoluble oxalate content of each sample was calculated by the difference between the total and soluble oxalate contents.

### 2.8. Standard Calibration

Two standard curves of oxalic acid (99.99% oxalic acid, Sigma-Aldrich Co., St. Louis, MO, USA) were analyzed using the following concentrations: 1, 2, 5, 10, 15 and 25 mg/100 mL, one prepared in 0.2 M HCl and the other prepared in high purity water. The acid standard curve was used for identifying and calculating the total oxalate content, while the water standard curve was used for calculating the soluble oxalate content. All blanks and standard solutions were passed through a 0.45 µm cellulose acetate filter prior to analysis. 

### 2.9. Mineral Analysis

Four replicates of the raw or cooked leaf samples were weighed into a tared Teflon microwave vessel, where the weight was recorded. 5 mL of 69% nitric acid (BDH Aristar, BDH Chemicals, Ltd., Poole, Dorset, UK) and 1 mL of 30% hydrogen peroxide (BDH Aristar, BDH Chemicals, Ltd., Poole, Dorset, UK) were then added. The Teflon PFA- and Kevlar-shielded vessels were then capped and digested using a CEM Mars Express microwave (CEM Corporation, Matthews, NC, USA). The heating program was as follows: ramp 10 min to 90 °C, hold for 5 min, ramp for 10 min to 150 °C, then hold for 5 min. The cooled digest was made up to 25 mL with high purity water (18.2 MΩ·cm, Barnstead International, Dubuque, IA, USA). 

The concentration of total calcium in each sample was measured on a Varian Axial 720 Inductively Coupled Plasma Optical Emission Spectrophotometer (ICP-OES, Varian, Palo Alto, CA, USA) with a SP3 auto-sampler. Minerals were identified and quantitated using an ICP multi-element standard solution (CertiPUR, Merck, KGaA, Darmstadt, Germany) containing 23 elements. The data and the standard curves were processed using ICP-Expert™ II (Varian, Palo Alto, CA, USA). A high purity water test standard and ICP multi-element standard solutions containing 0.50 μg/g of each element were run in triplicate to calibrate the ICP-OES instrument and to determine the standard error of the analyses. The overall standard error was ±0.013 mg/kg. A limit of quantitation (LOQ) test was performed using 10 times the standard deviation of the blank (5% *v/v* nitric acid) for each mineral. Individual mineral LOQ values ranged from 0.12 to 12.24 μg/L, with a mean of 1.81 μg/L.

### 2.10. Statistical Analysis

All calculations were performed using Excel 2013 (Microsoft, Redmond, WA, USA), and statistical analysis was carried out using GenStat, Release 15.1 (VSN International Ltd., Hemel Hempstead, Hertfordshire, UK) for Windows 7 (Microsoft, Redmond, WA, USA) to determine the accumulated analyses of variance. The mean values were compared using Duncan’s multiple range test (*p* < 0.05). All analyses were carried out in triplicate.

## 3. Results

### 3.1. Raw and Cooked Leaves

The total, soluble, and insoluble oxalate contents for the raw, boiled, and wok-fried fat hen leaves (on a dry weight basis) are shown in Table 3. Boiling the leaves for two min significantly reduced the total oxalate content of the cooked leaves, but the ratio of soluble oxalate to the total oxalate content remained the same as in the raw leaves (mean 75%).

Cooking the leaves in a wok for two min resulted increased dry matter content in the cooked leaves from the absorption of cooking oil (from 20.1 to 42.2 g/100 g wet matter (WM), respectively). The proportion of soluble oxalate content to the total oxalate content was reduced compared to the raw and boiled leaves, as a large proportion of soluble oxalate was converted into insoluble oxalate during wok-frying. The total calcium contents of the boiled leaves significantly increased when the leaves were boiled and significantly reduced in the wok-cooked leaves, as the leaves absorbed oil during cooking. 

### 3.2. Processed Raw Leaves—Pesto and Juice

The pesto was made from 22.3% fresh fat hen leaves together with the standard ingredients usually added to improve the consistency and taste of the final product. Fresh fat hen leaves made up 49.1% of the juice mix and, again, standard ingredients were added to make a pleasant tasting mix. During preparation, the masticating juicer removed a significant portion of plant fiber derived from the fresh leaves and other ingredients added to the mix. Seventy-five percent of the original leaves, fruits, and vegetables in the mix were recovered as juice (Table 2). The total, soluble, and insoluble oxalate contents for the pesto and juice (g/100 g juice) are shown in Table 4.

## 4. Discussion

The total calcium contents of the raw and cooked leaves were 224.60 ± 1.47 and 268.68 ± 3.62 mg/100 DM, respectively. This was low compared with the values 539.9 and 545.7 mg/100 g DM previously reported values for leaves of raw and cooked fat hen grown in Tamil Nadu, India [7]. Amariai and Pius [7] went on to measure the bioavailability of calcium using a simulated gastric equilibrium dialysis analysis after a simulated in vitro gastric digestion. Using this method, the mean bioavailability of calcium in the raw and boiled leaves grown in India was 32.9%. In this present study 65.69% and 78.14%, respectively, of the total calcium contents of the raw and boiled fat hen leaves were calculated to be soluble calcium. In contrast, the soluble calcium content of the wok-fried leaves was 20.78%, which corresponds to a marked increase in the proportion of insoluble oxalates in this fried food, which made up 46.6% of the total oxalate fraction (Table 3). The amount of soluble calcium calculated in this study is in contrast to those calculated for raw and cooked fat hen leaves prepared in India [7]. However, this study used very different methods to determine the soluble calcium fraction of the cooked leaves [7].

A similar mixture of ingredients was used to make a juice from three cultivars of bitter gourd fruits [14]. The mean recovery of juice from the mix was 75%, meaning that a significant amount of fiber was discarded by the masticating juicer. They also noted that the soluble oxalate contents the juices they prepared were very low, and the insoluble content was even lower. They commented that the other ingredients in the mixture would have effectively diluted the oxalate contents in the juice. In addition, the soluble and insoluble oxalates would tend to bind to the discarded fiber fraction. In this experiment, the total and soluble oxalate contents of the juice prepared (Table 4) were also relatively lower than in the raw fresh leaves for the same reason. It is interesting to note that the addition of a food source of calcium (dry powdered Parmesan cheese) to the pesto increased the insoluble oxalate content and, thus, decreased the available soluble oxalate, whereas the addition of calcium chloride to the juice had little effect. The addition of Parmesan cheese to the pesto mix had a similar effect as the addition of milk to taro leaves [10,11] and spinach leaves [12], while the addition of calcium chloride during the preparation of juice from fat hen leaves was not as effective as the addition of calcium chloride during the processing of rhubarb stems [9]. In this present experiment, it is possible that the addition of the other ingredients containing high fiber contents during the preparation of juice from fat hen leaves may have had the effect of reducing the overall soluble oxalate contents of the juice. While these added ingredients had the effect of reducing the overall soluble oxalate content, the effect of the addition of fiber—which the soluble oxalate may have bound to during juice preparation—possibly had the most important effect. This fiber was then removed in the discarded fiber fraction. The addition of calcium chloride to the juice was only marginally effective (Table 4). 

## 5. Conclusions

Overall, this experiment has shown that raw, boiled, and wok-fried fat hen leaves contained high levels of total and soluble oxalates. Boiling the leaves significantly reduced the soluble oxalate content by leaching, while the levels in wok-fried leaves were effectively diluted by the absorption of the salad oil into the leaves. The insoluble oxalate fraction in the wok-cooked leaves contained a significant proportion of the total calcium content, with the proportion of calcium locked in the insoluble oxalate ranging from 34 to 79% of the total calcium content in the leaves. Compared with raw leaves, the proportion of calcium locked in insoluble oxalate was reduced by boiling (34% vs. 22% of the total calcium content) and markedly increased by wok-frying (79%). This effect of wok-cooking decreases soluble oxalate availability for absorption but also decreases the availability of the calcium in the fat hen leaves. 

The soluble oxalate content of the pesto and juice made from the leaves was relatively low because the leaf content was reduced by the addition of the other fruits and vegetables used to make a balanced mix. These products would have little negative effect in the diet because of their low soluble oxalate content. In contrast, the leaves, if consumed regularly or in large amounts, could be of considerable risk in the diet due to absorption of soluble oxalate from the raw and boiled leaves. The reduced proportion of soluble oxalates in wok-cooked leaves because of binding to calcium within the leaves is advantageous as long as other adequate calcium sources are present in the diet. 

## Figures and Tables

**Table 1:** Recipe for fat hen pesto (g fresh weight).

Ingredients	Fresh Weight (g)
Fresh leaves	40
Tap water	60
Garlic cloves	20
Ground walnuts	20
Parmesan cheese ^1^	30
Olive oil	5
Salt	3
Pepper	1
Total weight of the pesto	179.0

^1^ Grated dried Parmesan cheese (Puhoi Valley Cheese, Puhoi, New Zealand), containing 1200 mg calcium/100 g DM.

**Table 2:** Recipe of the fat hen juice mixture (g fresh weight).

Ingredients	Fresh Weight (g)
Fresh leaves	140
Apples	40
Cucumber	40
Celery sticks	40
Lemon	25
Total weight of mix	285.0
Weight of juice recovered	212.9

**Table 3:** Mean dry matter, total, soluble, and insoluble oxalates, total calcium and calcium bound in insoluble oxalate content of fat hen leaves raw, boiled and wok-fried (mg/100 g DM ± SE).

Parameter	Raw	Boiled	Wok-Fried
Dry matter (g/100 g WM)	20.06 ± 0.31	11.58 ± 0.16	42.20 ± 1.16
Total oxalate (mg/100 g DM)	1112.40 ± 19.00 ^a^	682.79 ± 29.13 ^b^	883.57 ± 3.45 ^c^
Soluble oxalate (mg/100 g DM)	866.31 ± 14.87 ^a^	496.37± 7.37 ^b^	472.14 ± 10.83 ^b^
	(78.0)	(72.7)	(53.4)
Insoluble oxalate (mg/100 g DM)	246.09 ± 28.80 ^b^	186.42 ± 32.53 ^b^	411.43 ± 13.48 ^a^
Total calcium (mg100 g DM)	224.60 ± 1.47 ^b^	268.68 ± 3.62 ^a^	162.43 ± 1.23 ^c^
Calcium bound in insoluble oxalate	76.98 ± 9.01 ^b^	58.32 ± 19.18 ^b^	128.70 ± 4.21^c^
Soluble calcium/total calcium (%)	65.69 ± 4.13 ^b^	78.14 ± 4.08 ^a^	20.76 ± 2.58 ^c^

Values in brackets are % soluble oxalate in the total oxalate content. Means in each row with a different superscript letter are significantly different using Duncan’s multiple range test, *p* < 0.05.

**Table 4:** Dry matter, total, soluble, and insoluble oxalate contents of pesto (mg/100 g WM) and juice (g/100 g juice) made from raw fat hen leaves.

	Pesto	Juice	Juice + Calcium ^1^
Dry matter	30.89 ± 0.50	12.59 ± 0.05	12.59 ± 0.05
Total oxalate	196.25 ± 23.63	100.75 ± 3.94	100.75 ± 3.94
Soluble oxalate	58.50 ± 1.44	65.75 ± 1.11	60.50 ± 1.50
	(28.8)	(65.2)	(60.1)
Insoluble oxalate	138.0 ± 23.74	35.25 ± 4.59	40.75 ± 4.94

Values in brackets are % of soluble oxalate in the total oxalate content.^1^ 200 mg calcium chloride added to 100 mL of the juice.
